# Retraction Note: Heme oxygenase-1-modified bone marrow mesenchymal stem cells combined with normothermic machine perfusion to protect donation after circulatory death liver grafts

**DOI:** 10.1186/s13287-022-03200-8

**Published:** 2022-11-01

**Authors:** Huan Cao, Liu Yang, Bin Hou, Dong Sun, Ling Lin, Hong-Li Song, Zhong-Yang Shen

**Affiliations:** 1 Tianjin First Central Hospital Clinic Institute, Tianjin Medical University, Tianjin, 300070 People’s Republic of China; 2 Department of Organ Transplantation, Tianjin First Central Hospital, No. 24 Fukang Road, Nankai District, Tianjin, 300192 People’s Republic of China; 3 Tianjin Clinical Research Center for Organ Transplantation, Tianjin, People’s Republic of China; 4 NHC Key Laboratory of Critical Care Medicine, Tianjin, People’s Republic of China; 5 Tianjin Key Laboratory of Organ Transplantation, Tianjin, People’s Republic of China; 6 Key Laboratory of Transplant Medicine, Chinese Academy of Medical Sciences, Tianjin, People’s Republic of China

## Retraction to: Stem Cell Research & Therapy (2020) 11:218 https://doi.org/10.1186/s13287-020-01736-1

The Editors-in-Chief have retracted this article. After publication, concerns were raised regarding potential image overlap within the published figures. Specifically:

- In Fig. 4A, the HBP images for the Naive and 14 d groups appear to contain an overlapping area.
- In Fig. 5B, there appears to be overlap between the Sham-14 d and HBP-7 d, as well as NMP-1 d and BP-14 d images.
- In Fig. 9A, the western blot images representing MYD88-1 d and p-IκBα-7 d, as well as p-IκBα-Naive and p-p65-7 d appear highly similar.

The authors have stated that these errors occurred due to mishandling of the data. The Editors-in-Chief therefore no longer have confidence in the presented data.

All authors agree to this retraction.

